# Percutaneous injuries among dental professionals in Washington State

**DOI:** 10.1186/1471-2458-6-269

**Published:** 2006-10-30

**Authors:** Syed M Shah, Anwar T Merchant, James A Dosman

**Affiliations:** 1 Department of Community Health and Epidemiology, University of Saskatchewan, Saskatoon, Canada; 2 Canadian Centre for Health and safety in Agriculture, University of Saskatchewan, Saskatoon, Canada; 3 Safety & Health Assessment & Research for Prevention, (SHARP) Program, Washington State Department of Labor and Industries, Olympia, USA; 4 Department of Community Health and Epidemiology, McMaster University, Hamilton, Canada

## Abstract

**Background:**

Percutaneous exposure incidents facilitate transmission of bloodborne pathogens such as human immunodeficiency virus (HIV), hepatitis C virus (HCV) and hepatitis B virus (HBV). This study was conducted to identify the circumstances and equipment related to percutaneous injuries among dental professionals.

**Methods:**

We used workers' compensation claims submitted to the Department of Labor and Industries State Fund during a 7-year period (1995 through 2001) in Washington State for this study. We used the statement submitted by the injured worker on the workers' compensation claim form to determine the circumstances surrounding the injury including the type of activity and device involved.

**Results:**

Of a total of 4,695 accepted State Fund percutaneous injury claims by health care workers (HCWs), 924 (20%) were submitted by dental professionals. Out of 924 percutaneous injuries reported by dental professionals 894 (97%) were among dental health care workers in non-hospital settings, including dentists (66, 7%), dental hygienists (61, 18%) and dental assistants (667, 75%). The majority of those reporting were females (638, 71%). Most (781, 87%) of the injuries involved syringes, dental instruments (77, 9%), and suture needles (23%). A large proportion (90%) of injuries occurred in offices and clinics of dentists, while remainder occurred in offices of clinics and of doctors of medicine (9%), and a few in specialty outpatient facilities (1%). Of the 894 dental health care workers with percutaneous injuries, there was evidence of HBV in 6 persons, HCV in 30 persons, HIV in 3 persons and both HBV and HVC (n = 2) exposure.

**Conclusion:**

Out of hospital percutaneous injuries are a substantial risk to dental health professionals in Washington State. Improved work practices and safer devices are needed to address this risk.

## Background

Percutaneous injury is one of the major risk factors in the transmission of hepatitis C (HCV), hepatitis B (HBV) and human immunodeficiency virus (HIV) [1,2]. HCV is a leading cause of chronic hepatitis and cirrhosis [3], and to date there is no protective vaccine against HCV. Thus, it is crucial to develop an effective strategy to monitor and manage needlestick injuries (NSIs) among health care workers.

National and international guidelines, such as the Needlestick Safety Act in 2001 were developed to help minimize the risk of bloodborne pathogen exposure to health care workers including dental settings [4,5]. Needlestick injury rates declined after better compliance with infection control guidelines and more widespread use of safety-engineered devices in both teaching and non-teaching hospitals [6,7]. Hospitals also have been the focus of surveillance programs at the national level [8,9]. Most dental professionals work in small offices and clinics in the non-hospital settings, often with limited resources for injection safety and infection control. Few studies have been conducted to document the burden of the problem in these settings [10-12].

In this study we used the Washington State workers' compensation claims to identify circumstances and devices leading to percutaneous injuries among dentists, dental hygienists and dental assistants. These sources of data have been used successfully in several studies examining injury hazards and industries at risk [13-15].

## Methods

In Washington State, employers are required to obtain workers' compensation insurance through the Department of Labor and Industries (L&I) State Fund, unless they are self-insured, self-employed or employees of the federal government. The L&I State Fund covers approximately two-thirds of the workers in Washington State. The remainder are employed primarily by the 400 largest employers in Washington State and are covered by their self-insured employers' plan. Most federal workers are not included in L&I State Fund.

In this study we used workers' compensation claims during a 7-year period (1995 through 2001) in Washington State to study percutaneous injuries among dental professionals. We extracted data using two major workers' compensation data systems: the Labor and Industries' Industrial Insurance System (LINIIS), and the Medical Information Payment System (MIPS). The employer's industry was identified using the Standard Industrial Classification (SIC) coding system. We identified health care workers in the SIC 80 (health services) and the two major state funded teaching hospitals, classified under SIC 82 (educational services) with a date of injury between January 1, 1995, and December 31, 2001. Detailed analyses were restricted to dental health professionals working in non-hospital settings as there were few cases from hospitals. We extracted information on claimants' gender, age, and details of the injury and illness based on coding used by the American Standard Method of Measuring and Recording Injury Experience of the American National Standard Institute (ANSI) [16]. We identified percutaneous injury claims using ANSI source code, (e.g., source code 2202 for a needle and nature code 170 for sharp injury) or a text word search of the workers' compensation report of the accident form for percutaneous injuries. The principal investigator studied each claim in the electronic files of scanned and indexed claims databases also known as WISE (With Imaging Service Excellence) and the statement submitted by the injured worker on workers' compensation claim forms provided that required information about device and the circumstances of needle-stick injury. We estimated the total direct cost for the accepted NSI cases from data available from the workers medical bills. It represents only the reimbursement of medical costs associated with post-exposure tests, physician visits, and use of drugs. The injured worker had a known infection exposure if the source patient tests were positive for one of the major blood borne pathogens (HCV, HBV, HIV). Laboratory test results for HCV, HBV and HIV for both study subjects and source patients were available from the health care system as part of medical records in electronic form. Follow up data were not available for 332 source cases. We also noted hepatitis B vaccination status from injury reports and medical records. These injuries were accepted for workers' compensation for possibility of infection transmission. The workers compensation data provide a useful source to study needlesticks in non-hospital settings [17].

### Statistical Analysis

The analysis focused on L&I State Fund accepted NSI claims for dental health care workers in non-hospital settings. Descriptive analyses included frequency of claims by location in non-hospital settings, job category and year of study. Denominator data (work hours) were not available by occupation and we could not estimate rates and trends over the study period.

## Results

There were a total of 4,695 accepted State Fund percutaneous injury claims by health care workers (HCWs) of which 924 (20%) occurred among dental professionals. Out of 924 percutaneous injuries reported, 894 (97%) were reported by dental health care workers in non-hospital settings, including dentists (n = 66, 7%), dental hygienists (n = 161, 18%) and dental assistants (n = 667, 75%). The mean age was 30 years (95% CI: 29–31). The majority (n = 638, 71%) of the study participants were female. A large proportion (90%) of injuries occurred in offices and clinics of dentists and dentists in doctor's offices (9%), and a few in specialty outpatient facilities (1%). The absolute number of injuries reported increased progressively each year, from 78 in 1995 to 216 in 2001.

Among dental assistants, most injuries (N = 578, 86%) involved a syringe needle followed by dental instruments such as bur, explorer, scaler or scalpel (n = 60, 9%), a suture needle (n = 18, 3%) and other devices (n = 11, 2%). Among dental hygienists, most injuries were due to syringe needles (n = 147, 91%) followed by a dental instrument (n = 14, 9%). Among dentists, the majority of injuries were due to syringes (n = 54, 82%) followed by dental instruments (n = 7,11%) or suture needles (n = 5, 7%).

Out of 894 dental health care workers with percutaneous injuries follow-up data were not available for 332 source cases. Of those with follow-up data (n = 562, 63%), exposures to the source patient had evidence of blood-borne infections, including HCV (n = 30), HBV (n = 6) and HIV (n = 3) and 2 had multiple (HBV/HCV) infections. There were 3 seroconversions among dental professionals who were exposed to HCV.

Dental assistants sustained most (n = 160, 24%) of the injuries while cleaning instruments and trays, followed by changing a local anesthetic carpule (n = 125, 19%) and recapping a needle (n = 118, 18%). These activities led to most injuries (60%) as shown in Table 1. Dental hygienists experienced needle-stick injuries most frequently during preadministering of local anesthesia (24%), followed by recapping a needle (18%) and cleaning instruments and trays (14%). Administering local anesthesia, recapping a needle and performing surgical procedures were the most (70%) important causes of injuries among dentists.

The average direct workers' compensation cost per needle-stick injuries was $360. The average cost of claims involving a seropositive sources as HCV, HBV or HIV positive was $1,383. Immunization coverage for hepatitis B was 98% for dentists and dental hygienists, and 94% for dental assistants.

## Discussion

Using the local anesthetic syringe and recapping were the two most important causes of NSI in dentists and dental hygienists. Cleaning instruments, changing the anesthetic carpule, and recapping were the most common activities leading to percutaneous injuries in dental assistants.

Studies from US hospitals also indicated syringe use as the major cause of percutaneous injuries among dental professionals [9]. In dental practice multiple injection must be given over the course of the patient's treatment. These activities place dental professionals at an increased risk of sustaining needle stick injuries. There is a need for safer devices in such practices. Approximately 70% of U.S. hospitals have started using IV delivery systems that do not require the use of needles and the use of safer devices has diminished the risk posed to healthcare workers [7,18]. The Occupational Safety and Health Administration standards and updated CDC guidelines recommend safe work practices to avoid risky behavior [4,19]. Improved knowledge and training can reduce percutaneous injuries significantly [20,21].

To reduce NSI in the dental office there is a need to invest resources into educating employees on the proper use of devices, focusing on administration of local anesthetic, recapping, changing the anesthetic carpule and cleaning of instruments, as these factors contributed to a significant proportion of injuries among dental professionals in this study. Moreover, the training needs to be customized for the type of dental health care worker. The focus of training for dentists and dental hygienists could be prevention of percutaneous during local anesthetic administration, while for dental assistants it could be cleaning of instruments, and changing the anesthetic carpule. Recapping was a common cause of percutaneous in all three categories of dental heath care workers. Another possible strategy to prevent percutaneous injuries could be the re-engineering of the anesthetic needle so that it is less likely to cause injury. Although the benefits of this strategy would accrue in the future, they are likely to be more widespread.

One of the challenges in reaching dental health care workers is that this group is fragmented, often working in smaller places with limited resources for injection safety and infection control. However, as this is a group at risk of being exposed to and acquiring blood borne pathogens through needle-stick injuries, there is a need to develop innovative programming to address the problem, involving resources from government, industry, and professional organizations. We observed high immunization coverage (>94%) for hepatitis B among dental professionals. Infection rates for these pathogens have been on the decline over the past decade due to widespread immunization of healthcare workers for HBV [7].

The estimated direct costs associated with initial follow-up and treatment for a dental professional who sustained a percutaneous injury ranged from $360 to $1,383. Jagger et al. estimated that the average direct costs of initial treatment of NSI at two US hospitals were $672 and $539 per injury [22]. A single indicator such as direct cost, however, underestimates the true burden the disease placed on the individual. Exposure to blood borne pathogens via needle stick injuries exacts a significant emotional and psychological toll on the victims, the cost of which are difficult to measure [23,24].

We could not calculate rate of exposure by occupation, as denominator data were not available. However, in 2000 there were 5,670 dental hygienists and 8,420 dental assistants practicing in Washington State as compared with 43,500 nurses, but there were 828 reported needle-stick injuries in dental hygienists or dental assistants as compared with 1048 injuries among nurses between 1995 and 2001 [25]. These data suggest that dental health professionals may be at high risk of needle-stick injuries. The steady increase in the number of claims from dental health care professionals over the time period of this study could be due to a number of factors such as improved reporting, increased number of dental professionals (denominator), increased number of procedures and increase in risk.

The workers compensation data are collected for administrative purposes and not necessarily for research. A limitation of this study, which we could not control for, was that not all needle stick injuries were reported. The problem is further compounded when workers apply for workers' compensation coverage, the definition of an occupational disease may restrict whether or not the affected person qualifies for benefits. Studies show that between 9% and 45% of workers suffering occupational illness file for workers' compensation benefits [26]. The case definition of a needle-stick injury is sensitive to the ANSI z16.2 coding for type, source, and nature of injury claims. Our findings therefore underestimate the actual burden of NSI.

## Conclusion

Needlestick injuries are associated with a number of blood borne infections and are common among dental health professionals. The injuries are mainly related with cleaning instruments, recapping needles, and administering local anesthesia. Better training, care during cleaning instruments, avoiding hazardous practices such as recapping needles, and development of safer needles may prevent injury and disease.

## Competing interests

The author(s) declare that they have no competing interests.

## Authors' contributions

SMS was responsible for the over all planning and design of the research project, analysis, interpretation and writing up of the data. ATM and JAD were responsible for drafting and revising the manuscript for important intellectual content. All authors read and approved the final manuscript.

## Pre-publication history

The pre-publication history for this paper can be accessed here:



## Figures and Tables

**Table 1:** Mechanism of Reported Percutaneous Injuries Among Dental Professionals, Washington State, 1995–2001.

	Job Category		
Activity	Dental Assistant N (%)	Dental Hygienist N (%)	Dentist N (%)
Total Mechanism	667 (100.0%)	161 (100.0)	66 (100.0)
Cleaning Instrument	160 (24.0)	23 (14.3)	-
Changing carpule	125 (18.7)	13 (8.1)	5 (7.6)
Recapping	118 (17.7)	29 (18.0)	13 (19.7)
Cape fell off while removing needle from syrenge	57 (8.5)	6 (3,7)	4 (6.1)
Disposal related	36 (5.4)	3 (1.9)	2 (3.0)
Unknown	34 (5.1)	5 (3.1)	1 (1.5)
Dr hit	29 (4.3)	-	-
Collision	29 (4.3)	4 (2.5)	1 (1.5)
Preparing	23 (3.4)	6 (3.7)	1 (1.5)
Local anesthetic injection	21 (3.1)	39 (24.2)	22 (33.3)
Device left on table	11 (1.6)	1 (0.6)	-
Injecting other than a local	9 (1.3)	8 (5.0)	2 (3.0)
Unexpected jerk of patient	6 (0.9)	13 (8.1)	3 (4.5)
Dental cleaning	6 (0.9)	8 (5.0)	1 (1.5)
Surgery	3 (0.4)	-	11 (16.7)
Blood Draw	-	3 (1.9)	
